# Special Issue: Molecular Advance on Reproduction and Fertility of Aquatic Animals

**DOI:** 10.3390/ijms252111610

**Published:** 2024-10-29

**Authors:** Hui Qiao, Sufei Jiang, Hongtuo Fu

**Affiliations:** 1 Key Laboratory of Freshwater Fisheries and Germplasm Resources Utilization, Ministry of Agriculture and Rural Affairs, Freshwater Fisheries Research Center, Chinese Academy of Fishery Sciences, Wuxi 214081, China; qiaoh@ffrc.cn; 2 Wuxi Fisheries College, Nanjing Agricultural University, Wuxi 214081, China

## 1. Introduction

Many commercial aquatic animals are cultured in a variety of countries and regions. Reproduction and fertility are complex in aquatic animals. Sexual size dimorphism (SSD) or sexual shape dimorphism (SShD), are common phenomena in many commercial aquatic animals, with obvious differences between males and females [1,2,3]. Sexual dimorphism is more frequently identified in commercial aquatic species and has many important applications in commercial culture [4,5]. In some economic species, such as the tilapia (*Oreochromis* spp.) and giant freshwater prawn (*Macrobrachium rosenbergii*), males have greater growth rates (body weight and length) than females [6,7]. For other commercial species, such as the common carp (*Cyprinus carpio*), topmouth culter (*Culter alburnus*) and mud crab (*Scylla paramamosain*), females grow faster than males, with larger body sizes [8,9,10]. Thus, a monosexual culture (all-male or all-female) can greatly improve aquaculture efficiency and may have dramatic economic benefits [10,11,12,13,14,15], which in turn may bring about greater potential benefits in many commercial species by taking advantage of bisexual dimorphism. These benefits may include (1) faster growth; (2) controlling overbreeding and early sexual maturity; and (3) preventing the escape risk of impact on the environment [15]. In addition, both rapid and slow gonad development have negative effects on sustainable development. Slow gonad development will extend the breeding cycle [16,17], while rapid development will result in inbreeding between new-born animals, small size and low disease resistance [18,19]. Therefore, analyses of their reproductive mechanisms are equally important, and these should include but not be limited to molecular functional studies of sex differentiation and gonadal development. With the large-scale application of transcriptome sequencing technologies, many candidate genes involved in the molecular mechanisms of sex differentiation and gonadal development have been mined [20,21]. Fishes have a well-formed sex differentiation system and hypothalamic–pituitary–gonadal axis, and the relevant gene identification from brain and gonadal tissues helps to analyses processes of sexual differentiation and maturity [22,23,24,25]. The androgenic gland (AG), which is a distinctive endocrine organ in crustaceans, has been studied in depth with regard to sexual differentiation in males in many commercial species such as *Macrobrachium rosenbergii*, *Macrobrachium nipponense* and *Procambarus clarkii* [26,27,28]. In addition, environmental factors have been shown to have significant effects on aquatic animal reproduction and fertility, such as temperature, exogenous steroid hormones and biological or chemical reagents, etc. [29,30]. Aquatic animals have a multitude of sex determination mechanisms, ranging from genetic control (GSD) to environmental control (ESD), or even GSD-ESD interactions [31,32]. Several studies have proved that environmental factors, such as temperature [33,34,35], hypoxia [36,37,38] and endocrine-disrupting chemicals [39,40,41,42], have significant effects on sex determination and differentiation. A better understanding of the molecular mechanisms of reproduction in each species is fundamental to artificial breeding programmers.

This Special Issue, “Molecular Advances in Reproduction and Fertility of Aquatic Animals”, focuses on the following areas with regard to aquatic animals: genes or markers, as well as epigenetic modifications involved in sex differentiation, reproduction-related gene identification and gonadal maturation mechanisms under environmental risks of starvation. It provides an excellent collection of studies related to the hot topics of reproduction and fertility in aquatic animals, ranging from endogenous genetic mechanisms of sex differentiation and gonadal maturation to exogenous effects on gonadal maturation mechanisms due to nutritional factors.

## 2. Genes, Markers and Epigenetic Modifications Involved in the Sex Differentiation of Aquatic Animals

### 2.1. Sex-Differentiation-Related Genes and Markers in Pelodiscus sinensis

In the Chinese soft-shelled turtle *Pelodiscus sinensis*, sex is determined on the basis of a ZZ/ZW sex determination system, and exogenous hormones such as estradiol (E2) and methyltestosterone (MT) during sexual differentiation induce sex reversal in *P. sinensis* [43,44,45,46]. Sox3 is widely involved in the early regulation of neural stem cell differentiation [47,48] and plays an important role in male sex determination and gonadal differentiation in various species such as fish and amphibian species [49,50,51]. E2 and Sox3-RNAi treatment before sexual differentiation were administered in *P. sinensis*, and a transcriptomic analysis generated 1352, 908, 990, 1011 and 975 differentially expressed genes (DEGs) in five developmental stages, respectively, compared with treatment with only E2 [52]. The KEGG enrichment analysis of DEGs showed that Sox3 significantly affected sexual differentiation via the Wnt, TGF-β and TNF signaling pathways and mRNA surveillance pathway. The expression of Dkk4, Nog, Msi1 and Krt14 genes involved in the above signaling pathways changed significantly during gonadal differentiation. This article finally proves that Sox3 plays a catalytic role in the process of sex reversal and provides theoretical support for all-male breeding technology of *P. sinensis*.

An accurate and efficient workflow based on Python scripts was developed for the screening of sex-specific sequences with ZW or XY sex determination systems [53]. Based on this workflow, 4.01 Mb female-specific sequences were finally identified on *P. sinensis* reference genomes (female 2.23 Gb and male 2.26 Gb). A PCR genotyping method was established to verify the embryos’ and adults’ genetic sex, and the seven developed sex-specific primer pairs were 100% accurate in indicating that the embryos were genetically female and male, respectively. The studies also identified and functionally annotated many sex-determining candidate genes in female-specific genes and related pathways, including Ran, Eif4et and Crkl genes as well as the insulin signaling pathway and the cAMP signaling pathway. Finally, these findings highlight the strong potential of this workflow in the sex-specific sequence screening of *P. sinensis*. They also provide effective molecular tools for sex-controlled breeding and potential targets for studies of the sex-determination mechanisms in *P. sinensis*.

### 2.2. Lysosomal Acid Lipase (LIPA) Gene Regulates Sex Hormones and Inhibits Gonadal Development in Macrobrachium nipponense

In a previous sex determination mechanism study of *Macrobrachium nipponense*, the “steroid biosynthesis” pathway was examined using transcriptomic and KEGG enrichment analysis. A Lysosomal acid lipase (LIPA) gene in this pathway was suggested to play an important role in sex determination and gonadal differentiation [54,55]. LIPA catalyzes the hydrolysis of cholesterol esters or triglycerides that have been localized within lysosomes following the receptor-mediated endocytosis of low-density lipoprotein particles, and its functions have been determined in LIPA in many vertebrates [56]. However, there have been no reports on reproductive function. In *M. nipponense*, LIPA was proven to play an important role in sex hormone regulation and gonadal development for the first time [57]. It was highly expressed in the hepatopancreas, cerebral ganglion and testes, indicating its involvement in sex differentiation. ISH revealed LIPA signaling in the spermatheca and hepatopancreas, suggesting its role in steroid synthesis and sperm maturation. An RNAi Mn-LIPA increased the expression levels of male-specific genes, such as insulin-like androgenic gland hormone (IAG), sperm gelatinase (SG), and mab-3-related transcription factor (Dmrt11E) and sex hormone content (17β-estradiol and 17α-methyltestosterone). It also had a significant promoting effect on sperm development and maturation. These findings enhance our understanding of new functions of LIPA in sex differentiation and gonadal development and provide an important theoretical basis for the realization of a monosex culture of *M. nipponense*.

### 2.3. Functions of Epigenetic Modifications in the Sex Differentiation of Cyprinus carpio

Epigenetic modifications play important roles in sex determination and differentiation [58]. DNA methylation is the most extensively studied factor in epigenetic modification [59], while chromatin accessibility is the degree of physical contact between nuclear macromolecules and DNA, representing a type of significant epigenetic modification [60]. The common carp (*Cyprinus carpio*), which is a crucial fish cultivated worldwide, has an XX/XY sex-determination system, with females growing faster than males [61]. Monosex culture has great profit prospects for aquaculture industries. In a recent study, the authors firstly focused on the effects of DNA methylation and chromatin accessibility on subgenome expression dominance in the *C. carpio* [62]. The results indicated that gene expression patterns and functional characteristics were concordant across the two subgenomes in the common carp, and the relationship between DNA methylation and subgenome expression dominance needs to be further confirmed.

Furthermore, the authors performed ATAC-seq (Assay for Transposase Accessible Chromatin sequencing) and BS-seq (bisulfite sequencing) to explore the roles of epigenetic modifications in common carp gonads [63]. In total, 84,207 more accessible regions and 77,922 less accessible regions in ovaries compared to testes were identified and some sex-biased genes showed differential chromatin accessibility in their promoter regions, such as sox9a and zp3. The embryonic-development- and cell-proliferation-associated transcription factors (TFs) were mainly enriched in the ovaries, and the TFs Foxl2 and SF1 were only identified in the ovaries. In total, 5264 gene body differentially methylated genes (genebody-DMGs) in CG contexts were identified, and these were significantly enriched in the Wnt signaling pathway, TGF-beta signaling pathway, and GnRH signaling pathway. These results showed that methylation in gene body regions may play an essential role in sex maintenance. They also revealed that the expression of dmrtb1-like, spag6, and fels was negatively correlated with their methylation levels in promoter regions. To the best of our knowledge, this is the first time that the functions of epigenetic modification have been studied in common carp gonads. The results contribute to elucidating the molecular mechanisms of sex differentiation and sex maintenance in fishes.

## 3. Identification of Reproduction-Related Genes in Aquatic Animals

### 3.1. Dynein Intermediate Chain and Lamin B Were Involved in the Spermatogenesis of Portunus trituberculatus

Spermatogenesis in mammals normally includes acrosome formation, nucleus shaping and tail formation, and the mature sperm have a head and tail [64]. In contrast, the decapod crustacean sperm formation lacks tail formation, and the sperm nuclei are unconcentrated [65]. Lamins are components of the nuclear lamina, and the Lamin B gene has been suggested to maintain nuclear morphology and function during spermatogenesis in mammals and insects [65,66]. In previous studies, the Dynein intermediate chain (DIC) gene has been proven to play an important role in the nuclear deformation of spermiogenesis in *P. trituberculatus* [67]. In further studies [68], the authors give a next step hypothesis that Lamin B and DIC may have an interaction between them which is involved in the spermiogenesis process of *P. trituberculatus*. Subsequently, they obtained the full length of Lamin B and found that its protein distributes during spermatogenesis, which suggests that it is involved in nuclear deformation and acrosome formation. Coimmunoprecipitation further demonstrated that the DIC and Lamin B protein were involved in the spermiogenesis process. After RNAi of DIC, the distribution of both proteins in spermatogenesis was abnormal, and the abnormal proportion of spermatid nucleus deformation in the middle stage significantly increased. This study provides a theoretical basis for revealing the mechanisms of spermatogenesis in decapod crustaceans.

### 3.2. Vitellogenin Gene Family Involved in Ovarian Maturation in Exopalaemon carinicauda

Vitellogenin (Vtg) is the yolk source for ovary and embryo development in all oviparous organisms [69,70]. Vitellogenesis, which is the yolk synthesis and accumulation process in oocytes, is crucial in the reproduction of oviparous species [71]. The Vtg gene family has many paralogs, displaying differences in the structure and quantity and playing various roles in ovary and embryo development [72]. Understanding the roles of Vtgs in vitellogenesis processing is a benefit for the regulation of ovarian and embryo development in aquaculture. In *Exopalaemon carinicauda*, 10 Vtg genes were identified and characterized from the genomes [73]. These Vtgs were unevenly distributed on the chromosomes, indicating that they were constrained by purifying selection. All these Vtgs were expressed much higher in the female hepatopancreas than in other tissues, and their expression patterns during ovarian development indicated that the hepatopancreas is the main synthesis site. Among these Vtgs, Vtg1a and Vtg2 play major roles in exogenous vitellogenesis, while Vtg3 plays a major role in both exogenous and endogenous vitellogenesis. Vtgs expression in the female hepatopancreas can be significantly upregulated by the bilateral ablation of the eyestalk, suggesting that the X-organ/sinus gland complex is involved in regulating ovarian development. These results provide a basis for improving our understanding of the evolutionary and biological functions of Vtg genes in crustaceans.

## 4. Gonad Maturation Mechanism Under Environmental Risk of Starvation

With regard to environmental factors, the availability of food resources also has significant effects on reproductive processes of aquatic animals, affecting factors such as maturity and egg size, the number of broods, fecundity, ovarian development and vitellogenesis [74]. Previous studies in aquatic invertebrates [75] have shown that the basal maintenance functions are typically prioritized, and growth, reproduction, and activity are reduced during food limitation. A short starvation period could modify the lipid and protein contents in the ovary of *Penaeus monodon* [76] and stimulate oogenesis in drosophila [77]. Mud crabs (*Scylla paramamosain*) were starved for short-term and long-term periods in this study [78]. Histological and biochemical analysis, as well as transcriptomic analysis, were performed in the hepatopancreas, ovary, and serum to measure the dynamics of tissue structures, material compositions, and metabolic changes. The ovary structure, fatty acid compositions, and serum biochemistry underwent adaptive changes in a two-step process under starvation conditions, with hepatopancreas fatty acid supply to the ovary from day 7 to day 14 and autophagy activation in both organs from day 28 to day 40. The transcriptomic results generated candidate gene modules notably linked to physiological traits. Finally, the authors provide their ovarian development strategy hypothesis that (1) higher amounts of fatty acid are stored in the hepatopancreas for nutrient supply and (2) autophagy-related pathways are activated for terminal investment in reproduction. The results for *S. paramamosain* in this study provide a reference for adaptation mechanism research in response to starvation during ovarian maturation in other crustaceans. A better understanding of the relationship between autophagy and ovarian maturation will give new ideas for improving the nutritional and economic value of female crustaceans under starvation stress.

Overall, this Special Issue, “Molecular Advances in Reproduction and Fertility of Aquatic Animals”, provides a diverse collection of research articles covering hot topics of reproduction and fertility of aquatic animals. The genetic mechanisms of sex differentiation and gonadal maturation have been illustrated in depth by the functional analysis of related genes or markers and the impact analysis of epigenetic modification and environmental starvation. Notably, a deeper understanding of the molecular mechanisms of reproduction and fertility in aquatic animals depends on advances in various ‘omic’ sequencing technologies, such as genomics, transcriptomics, epigenomics and proteomics [79]. Among the candidate genes screened in these ‘omic’ sequencing explorations, the identification of the key major genes and their functions is a hotspot for further research. In the future, recent technological advances in haplotype-resolved genome assembly and genome editing will help to illustrate genetic mechanisms of sex differentiation and gonadal maturation [80,81,82]. Accurate sex and reproductive artificial control can enable famers to achieve monosex products and greater growth traits via molecular genetic techniques, even in nonbreeding conditions [83]. The detail mechanisms of GSD-ESD interaction can increase the efficiency of artificial sex control techniques by using environmental control [84].

## References

[1] Tan K., Yu J., Liao S., Huang J., Li M., Wang W. (2022). Transcriptomic profiling and novel insights into the effect of AG ablation on gonad development in *Macrobrachium rosenbergii*. Aquaculture.

[2] Paschoal L.R.P., Jos’e Zara F. (2019). The androgenic gland in male morphotypes of the Amazon River prawn *Macrobrachium amazonicum* (Heller, 1862). Gen. Comp. Endocrinol..

[3] Dutney L., Elizur A., Lee P. (2017). Analysis of sexually dimorphic growth in captive reared cobia (*Rachycentron canadum*) and the occurrence of intersex individuals. Aquaculture.

[4] Mei J., Gui J.F. (2015). Genetic basis and biotechnological manipulation of sexual dimorphism and sex determination in fish. Sci. China Life Sci..

[5] Benetti D.D., Sardenberg B., Hoenig R., Welch A., Stieglitz J., Miralao S., Farkas D., Brown P., Jory D. (2010). Cobia (*Rachycentron canadum*) hatchery-to-market aquaculture technology: Recent advances at the University of Miami Experimental Hatchery (UMEH). Rev. Bras. Zootec..

[6] Aflalo E.D., Hoang T.T.T., Nguyen V.H., Lam Q., Nguyen D.M., Trinh Q.S., Raviv S., Sagi A. (2006). A novel two-step procedure for mass production of all-male populations of the giant freshwater prawn *Macrobrachium rosenbergii*. Aquaculture.

[7] Lind C.E., Safari A., Agyakwah S.K., Attipoe F.Y.K., El-Naggar G.O., Hamzah A., Ponzoni R.W. (2015). Differences in sexual size dimorphism among farmed tilapia species and strains undergoing genetic improvement for body weight. Aquac. Rep..

[8] Ma H.Y., Ma C.Y., Ma L.B., Xu Z., Feng N.N., Qiao Z.G. (2013). Correlation of growth related traits and their effects on body weight of the mud crab (*Scylla paramamosain*). Genet. Mol. Res..

[9] Wang M., Chen L., Zhou Z., Xiao J., Chen B., Huang P., Li C., Xue Y., Liu R., Bai Y. (2023). Comparative transcriptome analysis of early sexual differentiation in the male and female gonads of common carp (*Cyprinus carpio*). Aquaculture.

[10] Cheng S., Chi M.L., Liu S.L., Zheng J.B., Jiang W.P., Hang X.Y., Peng M., Li F. (2024). Study on hormone induction of the male parent and embryonic development, gonad differentiation, and growth of “All-female No. 1” *Culter alburnus*. Heliyon.

[11] Tessema A., Getahun A., Mengistou S., Fetahi T., Dejen E. (2020). Reproductive biology of common carp (*Cyprinus carpio* Linnaeus, 1758) in Lake Hayq, Ethiopia. Fish. Aquat. Sci..

[12] Zhai G., Shu T., Chen K., Lou Q., Jia J., Huang J., Shi C., Jin X., He J., Jiang D. (2022). Successful production of an all-female common carp (*Cyprinus carpio* L.) population using cyp17a1-deficient neomale carp. Engineering.

[13] Fernandino J.I., Hattori R.S. (2019). Sex determination in neotropical fish: Implications ranging from aquaculture technology to ecological assessment. Gen. Comp. Endocrinol..

[14] Wahl M., Levy T., Ventura T., Sagi A. (2023). Monosex populations of the giant freshwater prawn *Macrobrachium rosenbergii*—From a pre-molecular start to the next generation era. Int. J. Mol. Sci..

[15] Basavaraju Y. (2023). Monosex Population in Aquaculture. Frontiers in Aquaculture Biotechnology.

[16] Wan G., Zhang H., Wang P., Qin Q., Zhou X., Xiong G., Wang X., Hu Y. (2024). Gonadal Transcriptome Analysis Reveals that SOX17 and CYP26A1 are involved in sex differentiation in the Chinese soft-shelled turtle (*Pelodiscus sinensis*). Biochem. Genet..

[17] Zhang D., Xu S., Wang Y., Wang Y., Ruan Z., Zhang C., Ren A., Xue B., Chai X. (2024). The development of gonads and changes of gonadotropin in silver pomfret *Pampus argenteus*. Thalassas.

[18] Liu P., Zhao X., Tang Q., Li J., Xia Z., Dong H., Yang G., Yi S., Gao Q. (2024). Transcriptome analysis of the gonad reveals growth differences between large, medium and small individuals in a pure family of *Macrobrachium rosenbergii*. Aquaculture.

[19] Jiang S., Xie Y., Gao Z., Niu Y., Ma C., Zhang W., Fu H. (2024). Studies on the relationships between growth and gonad development during first sexual maturation of *Macrobrachium nipponense* and associated SNPs screening. Int. J. Mol. Sci..

[20] Yano A., Guyomard R., Nicol B., Jouanno E., Quillet E., Klopp C., Cabau C., Bouchez O., Fostier A., Guiguen Y. (2012). An immune-related gene evolved into the master sex-determining gene in rainbow trout, *Oncorhynchus mykiss*. Curr. Biol..

[21] Tao W., Chen J., Tan D., Yang J., Sun L., Wei J., Conte M.A., Kocher T.D., Wang D. (2018). Transcriptome display during tilapia sex determination and differentiation as revealed by RNA-Seq analysis. BMC Genom..

[22] Chapman R.W., Reading B.J., Sullivan C.V. (2014). Ovary transcriptome profiling via artificial intelligence reveals a transcriptomic fingerprint predicting egg quality in striped bass, *Morone saxatilis*. PLoS ONE.

[23] Roy A., Basak R., Rai U. (2017). De novo sequencing and comparative analysis of testicular transcriptome from different reproductive phases in freshwater spotted snakehead *Channa punctatus*. PLoS ONE.

[24] Bar I., Cummins S., Elizur A. (2016). Transcriptome analysis reveals differentially expressed genes associated with germ cell and gonad development in the Southern bluefin tuna (*Thunnus maccoyii*). BMC Genom..

[25] Jin S.B., Zhang Y., Dong X.L., Xi Q.K., Song D., Fu H.T. (2015). Comparative transcriptome analysis of testes and ovaries for the discovery of novel genes from Amur sturgeon (*Acipenser schrenckii*). Genet. Mol. Res..

[26] Jin S., Fu H., Zhou Q., Sun S., Jiang S., Xiong Y. (2013). Transcriptome analysis of androgenic gland for discovery of novel genes from the oriental river prawn, *Macrobrachium nipponense*, using Illumina Hiseq 2000. PLoS ONE.

[27] Jiang H., Xing Z., Lu W., Qian Z., Yu H., Li J. (2014). Transcriptome analysis of red swamp crawfish *Procambarus clarkii* reveals genes involved in gonadal development. PLoS ONE.

[28] Levy T., Zupo V., Mutalipassi M., Somma E., Ruocco N., Costantini M., Abehsera S., Manor R., Chalifa-Caspi V., Sagi A. (2021). Protandric transcriptomes to uncover parts of the crustacean sex-differentiation puzzle. Front. Mar. Sci..

[29] Devlin R.H., Nagahama Y. (2002). Sex determination and sex differentiation in fish: An overview of genetic, physiological, and environmental influences. Aquaculture.

[30] Wilhelm D., Palmer S., Koopman P. (2007). Sex determination and gonadal development in mammals. Physiol. Rev..

[31] Budd A., Banh Q., Domingos J., Jerry D. (2015). Sex control in fish: Approaches, challenges and opportunities for aquaculture. J. Mar. Sci. Eng..

[32] Godwin J., Luckenbach J.A., Borski R.J. (2003). Ecology meets endocrinology: Environmental sex determination in fishes. Evol. Dev..

[33] Luzio A., Matos M., Santos D., Fontainhas-Fernandes A.A., Monteiro S.M., Coimbra A.M. (2016). Disruption of apoptosis pathways involved in zebrafish gonad differentiation by 17α-ethinylestradiol and fadrozole exposures. Aquat. Toxicol..

[34] Brown A.R., Owen S.F., Peters J., Zhang Y., Soffker M., Paull G.C., Hosken D.J., Wahab M.A., Tyler C.R. (2015). Climate change and pollution speed declines in zebrafish populations. Proc. Natl. Acad. Sci. USA.

[35] Abozaid H., Wessels S., Horstgen-Schwark G. (2012). Elevated temperature applied during gonadal transformation leads to male bias in zebrafish (*Danio rerio*). Sex. Dev..

[36] Shang E.H., Yu R.M., Wu R.S. (2006). Hypoxia affects sex differentiation and development, leading to a male-dominated population in zebrafish (*Danio rerio*). Environ. Sci. Technol..

[37] Robertson C.E., Wright P.A., Koblitz L., Bernier N.J. (2014). Hypoxia-inducible factor-1 mediates adaptive developmental plasticity of hypoxia tolerance in zebrafish, *Danio rerio*. Proc. Biol. Sci..

[38] Baroiller J.F., D’Cotta H. (2016). The reversible sex of gonochoristic fish: Insights and consequences. Sex. Dev..

[39] Caspillo N.R., Volkova K., Hallgren S., Olsson P.E., Porsch-Hallstrom I. (2014). Shortterm treatment of adult male zebrafish (*Danio rerio*) with 17α-ethinylestradiol affects the transcription of genes involved in development and male sex differentiation. Comp. Biochem. Physiol. C Toxicol. Pharmacol..

[40] Van der Ven L.T., van den Brandhof E.J., Vos J.H., Wester P.W. (2007). Effects of the estrogen agonist 17β-estradiol and antagonist tamoxifen in a partial life-cycle assay with zebrafish (*Danio rerio*). Environ. Toxicol. Chem..

[41] Tokumoto T., Tokumoto M., Nagahama Y. (2005). Induction and inhibition of oocyte maturation by EDCs in zebrafish. Reprod. Biol. Endocrinol..

[42] Xu H., Yang J., Wang Y., Jiang Q., Chen H., Song H. (2008). Exposure to 17α-ethynylestradiol impairs reproductive functions of both male and female zebrafish (*Danio rerio*). Aquat. Toxicol..

[43] Zhou T., Sha H., Chen M., Chen G., Zou G., Liang H. (2021). MicroRNAs may play an important role in sexual reversal process of Chinese soft-shelled turtle, *Pelodiscus sinensis*. Genes.

[44] Liang H.W., Cao L.H., Li X., Tong M.M., Jiang Y.L., Li Z., Luo X.Z., Zou G.W. (2017). Morphological differences analysis of three strains of *Pelodiscus sinensis*. Freshw. Fish..

[45] Kawagoshi T., Uno Y., Matsubara K., Matsuda Y., Nishida C. (2009). The ZW micro-sex chromosomes of the Chinese soft-shelled turtle (*Pelodiscus sinensis*, Trionychidae, Testudines) have the same origin as chicken chromosome 15. Cytogenet. Genome Res..

[46] Mu Y., Zhao B., Tang W.Q., Sun B.J., Zeng Z.G., Valenzuela N., Du W.G. (2015). Temperature-dependent sex determination ruled out in the Chinese soft-shelled turtle (*Pelodiscus sinensis*) via molecular cytogenetics and incubation experiments across populations. Sex. Dev..

[47] Topalovic V., Krstic A., Schwirtlich M., Dolfini D., Mantovani R., Stevanovic M., Mojsin M. (2017). Epigenetic regulation of human SOX3 gene expression during early phases of neural differentiation of NT2/D1 cells. PLoS ONE.

[48] Wang T.W., Stromberg G.P., Whitney J.T., Brower N.W., Klymkowsky M.W., Parent J.M. (2006). Sox3 expression identifies neural progenitors in persistent neonatal and adult mouse forebrain germinative zones. J. Comp. Neurol..

[49] Oshima Y., Naruse K., Nakamura Y., Nakamura M. (2009). Sox3: A transcription factor for Cyp19 expression in the frog *Rana rugosa*. Gene.

[50] Takehana Y., Matsuda M., Myosho T., Suster M.L., Kawakami K., Shin I.T., Kohara Y., Kuroki Y., Toyoda A., Fujiyama A. (2014). Co-option of Sox3 as the male-determining factor on the Y chromosome in the fish Oryzias dancena. Nat. Commun..

[51] Yao B., Zhou L., Wang Y., Xia W., Gui J.F. (2007). Differential expression and dynamic changes of SOX3 during gametogenesis and sexreversal in protogynous hermaphroditic fish. J. Exp. Zool. A Ecol. Genet. Physiol..

[52] Zhou T., Cao J., Chen G., Wang Y., Zou G., Liang H. (2024). Role of Sox3 in Estradiol-Induced Sex Reversal in *Pelodiscus sinensis*. Int. J. Mol. Sci..

[53] Zhu J., Wang Y., Chen C., Ji L., Hong X., Liu X., Chen H., Wei C., Zhu X., Li W. (2024). Identification of sex-specific markers and candidate genes using WGS sequencing reveals a ZW-type sex-determination system in the Chinese soft-shell turtle (*Pelodiscus sinensis*). Int. J. Mol. Sci..

[54] Cai P., Yuan H., Gao Z., Qiao H., Zhang W., Jiang S., Xiong Y., Gong Y., Wu Y., Jin S. (2023). 17β-estradiol induced sex reversal and gonadal transcriptome analysis in the oriental river prawn (*Macrobrachium nipponense*): Mechanisms, pathways, and potential Harm. Int. J. Mol. Sci..

[55] Reed K., Cronan J. (1993). Lipoic acid metabolism in Escherichia coli: Sequencing and functional characterization of the lipA and lipB genes. J. Bacteriol..

[56] Morris G., Braund P., Moore J., Samani N., Codd V., Webb T. (2017). Coronary artery disease–associated LIPA coding variant rs1051338 reduces lysosomal acid lipase levels and activity in lysosomes. Arterioscler. Thromb. Vasc. Biol..

[57] Cai P., Zhang W., Jiang S., Xiong Y., Qiao H., Yuan H., Gao Z., Zhou Y., Jin S., Fu H. (2024). Role of Mn-LIPA in sex hormone regulation and gonadal development in the oriental river prawn, *Macrobrachium nipponense*. Int. J. Mol. Sci..

[58] Ge C., Ye J., Weber C., Sun W., Zhang H., Zhou Y., Cai C., Qian G., Capel B. (2018). The histone demethylase KDM6B regulates temperature-dependent sex determination in a turtle species. Science.

[59] Ortega-Recalde O., Goikoetxea A., Hore T.A., Todd E.V., Gemmell N.J. (2020). The genetics and epigenetics of sex change in fish. Annu. Rev. Anim. Biosci..

[60] Klemm S.L., Shipony Z., Greenleaf W.J. (2019). Chromatin accessibility and the regulatory epigenome. Nat. Rev. Genet..

[61] Feng X., Yu X., Fu B., Wang X., Liu H., Pang M., Tong J. (2018). A high-resolution genetic linkage map and QTL fine mapping for growth-related traits and sex in the Yangtze River common carp (*Cyprinus carpio haematopterus*). BMC Genom..

[62] Yu S.T., Zhao R., Sun X.Q., Hou M.X., Cao Y.M., Zhang J., Chen Y.J., Wang K.K., Zhang Y., Li J.T. (2024). DNA methylation and chromatin accessibility impact subgenome expression dominance in the common carp (*Cyprinus carpio*). Int. J. Mol. Sci..

[63] Hou M., Wang Q., Zhao R., Cao Y., Zhang J., Sun X., Yu S., Wang K., Chen Y., Zhang Y. (2024). Analysis of chromatin accessibility and DNA methylation to reveal the functions of epigenetic modifications in *Cyprinus carpio* gonads. Int. J. Mol. Sci..

[64] Nishimura H., L’Hernault S.W. (2017). Spermatogenesis. Curr. Biol..

[65] Broers J.L., Machiels B.M., Kuijpers H.J., Smedts F., van den Kieboom R., Raymond Y., Ramaekers F.C. (1997). A- and B-type lamins are differentially expressed in normal human tissues. Histochem. Cell Biol..

[66] Melcer S., Gruenbaum Y., Krohne G. (2007). Invertebrate lamins. Exp. Cell Res..

[67] Wei C.G., Mu D.L., Tang D.J., Zhu J.Q., Hou C.C. (2021). Expression and functional analysis of cytoplasmic dynein during spermatogenesis in *Portunus trituberculatus*. Cell Tissue Res..

[68] Wang S.Y., Xiang Q.M., Zhu J.Q., Mu C.K., Wang C.L., Hou C.C. (2024). The functions of Pt-DIC and Pt-Lamin B in spermatogenesis of *Portunus trituberculatus*. Int. J. Mol. Sci..

[69] Carducci F., Biscotti M.A., Canapa A. (2019). Vitellogenin gene family in vertebrates: Evolution and functions. Eur. Zool. J..

[70] Sun C., Zhang S. (2015). Immune-relevant and antioxidant activities of vitellogenin and yolk proteins in fish. Nutrients.

[71] Dietrich M.A., Adamek M., Teitge F., Teich L., Jung-Schroers V., Malinowska A., Świderska B., Rakus K., Kodzik N., Chadzińska M. (2022). Proteomic analysis of carp seminal plasma provides insights into the immune response to bacterial infection of the male reproductive system. Fish Shellfish Immunol..

[72] Li L., Li X.J., Wu Y.M., Yang L., Li W., Wang Q. (2017). Vitellogenin regulates antimicrobial responses in Chinese mitten crab, *Eriocheir sinensis*. Fish Shellfish Immunol..

[73] Wang J., Tang S., Ge Q., Wang Q., He Y., Ren X., Li J., Li J. (2024). Genome-wide identification of vitellogenin gene family and comparative analysis of their involvement in ovarian maturation in *Exopalaemon carinicauda*. Int. J. Mol. Sci..

[74] Donelson J.M., Munday P.L., McCormick M.I., Pankhurst N.W., Pankhurst P.M. (2010). Effects of elevated water temperature and food availability on the reproductive performance of a coral reef fish. Mar. Ecol. Prog. Ser..

[75] Sokolova I.M., Frederich M., Bagwe R., Lannig G., Sukhotin A.A. (2012). Energy homeostasis as an integrative tool for assessing limits of environmental stress tolerance in aquatic invertebrates. Mar. Environ. Res..

[76] Kawabata T., Yoshimori T. (2016). Beyond starvation: An update on the autophagic machinery and its functions. J. Mol. Cell. Cardiol..

[77] Chang Y.Y., Neufeld T.P. (2010). Autophagy takes flight in Drosophila. FEBS Lett..

[78] Fu Y., Zhang F., Wang W., Xu J., Zhao M., Ma C., Cheng Y., Chen W., Su Z., Lv X. (2024). Temporal and spatial signatures of *Scylla paramamosain* transcriptome reveal mechanistic insights into endogenous ovarian maturation under risk of starvation. Int. J. Mol. Sci..

[79] Waiho K., Ikhwanuddin M., Afiqah-Aleng N., Shu-Chien A.C., Wang Y., Ma H., Fazhan H. (2022). Transcriptomics in advancing portunid aquaculture: A systematic review. Rev. Aquacult..

[80] Ramos L., Antunes A. (2022). Decoding sex: Elucidating sex determination and how high-quality genome assemblies are untangling the evolutionary dynamics of sex chromosomes. Genomics.

[81] Rhie A., Nurk S., Cechova M., Hoyt S.J., Taylor D.J. (2023). The complete sequence of a human Y chromosome. Nature.

[82] Ansai S., Kitano J. (2022). Speciation and adaptation research meets genome editing. Philos. Trans. R. Soc. B.

[83] Kitano J., Ansai S., Takehana Y., Yamamoto Y. (2024). Diversity and convergence of sex-determination mechanisms in teleost fish. Annu. Rev. Anim. Biosci..

[84] Toyota K. (2024). Crustacean endocrinology: Sexual differentiation and potential application for aquaculture. Gen. Comp. Endocr..

